# Nanomedicines in the Management of Alzheimer’s Disease: Current View and Future Prospects

**DOI:** 10.3389/fnagi.2022.879114

**Published:** 2022-07-08

**Authors:** Hitesh Chopra, Shabana Bibi, Inderbir Singh, Mohammad Amjad Kamal, Fahadul Islam, Fahad A. Alhumaydhi, Talha Bin Emran, Simona Cavalu

**Affiliations:** ^1^Chitkara College of Pharmacy, Chitkara University, Punjab, India; ^2^Department of Biosciences, Shifa Tameer-e-Millat University, Islamabad, Pakistan; ^3^Yunnan Herbal Laboratory, College of Ecology and Environmental Sciences, Yunnan University, Kunming, China; ^4^Institutes for Systems Genetics, Frontiers Science Center for Disease-Related Molecular Network, West China Hospital, Sichuan University, Chengdu, China; ^5^King Fahd Medical Research Center, King Abdulaziz University, Jeddah, Saudi Arabia; ^6^Department of Pharmacy, Faculty of Allied Health Sciences, Daffodil International University, Dhaka, Bangladesh; ^7^Enzymoics, Novel Global Community Educational Foundation, Hebersham, NSW, Australia; ^8^Department of Medical Laboratories, College of Applied Medical Sciences, Qassim University, Buraydah, Saudi Arabia; ^9^Department of Pharmacy, BGC Trust University Bangladesh, Chittagong, Bangladesh; ^10^Faculty of Medicine and Pharmacy, University of Oradea, Oradea, Romania

**Keywords:** Alzheimer’s disease, neurodegenerative disorders, nanotechnology, nanomedicine, dementia, aging

## Abstract

Alzheimer’s disease (AD) is a kind of dementia that creates serious challenges for sufferers’ memory, thinking, and behavior. It commonly targeting the aging population and decay the brain cells, despite attempts have been performed to enhance AD diagnostic and therapeutic techniques. Hence, AD remains incurable owing to its complex and multifactorial consequences and still there is lack of appropriate diagnostics/therapeutics option for this severe brain disorder. Therefore, nanotechnology is currently bringing new tools and insights to improve the previous knowledge of AD and ultimately may provide a novel treatment option and a ray of hope to AD patients. Here in this review, we highlighted the nanotechnologies-based findings for AD, in both diagnostic and therapeutic aspects and explained how advances in the field of nanotechnology/nanomedicine could enhance patient prognosis and quality of life. It is highly expected these emerging technologies could bring a research-based revolution in the field of neurodegenerative disorders and may assist their clinical experiments and develop an efficacious drug for AD also. The main aim of review is to showcase readers the recent advances in nanotechnology-based approaches for treatment and diagnosing of AD.

## Introduction

Alzheimer’s disease (AD) is frequently linked to memory loss and other critical cognitive abilities (Anand et al., 2014). The most common cause of dementia in the world is AD (Alzheimer’s Association, 2022). Although growing older is an established risk factor, AD is not a natural aspect of becoming older. Since the symptoms of AD grow with time, it’s important to monitor progress (Soliman et al., 2021; Walia et al., 2021). Although there is presently no cure for the condition, it may be slowed down by the use of medicines. AD is the result of a complex interplay of factors. The pathogenesis of this disease is still being re-examined (Tran and Ha-Duong, 2015). Cortical atrophy and the loss of neurons in the parietal and temporal lobes are hallmarks of AD (Walia et al., 2021). The brain’s ventricles also grow in size when brain mass diminishes (Tiwari et al., 2019). Slowly, the person’s cognition alters as a result of brain tissue alterations.

Patients with Alzheimer’s have higher quantities of extracellular amyloid in their brains compared to those with normal aging (Liu et al., 2015a). Neocortical and amygdala neurofibrillary tangles and nucleus basalis of meynert neurofibrillary tangles have also been reported (Serrano-Pozo et al., 2011). Beta-amyloid (Aβ) is dissolved and eliminated from the brain in a healthy brain. The Aβ protein may fold in on itself if it is not reabsorbed. Plaques are formed as a result of the proteins interacting with one another. More brain tissue is damaged as a consequence of the inflammatory response caused by these plaques (Reitz and Mayeux, 2014). Thalamus, dorsal tegmentum, locus ceruleus, paramedian reticular region, and the hypothalamus lateral nuclei may all be affected (Reitz and Mayeux, 2014).

Reduced choline acetyltransferase activity in the cerebral cortex and hippocampus, besides the death of cholinergic neurons along the cholinergic projection route to the hippocampus, are thought to be the root causes of these degenerative changes (Reitz and Mayeux, 2014). Above seventy-five percent of the patients here are over the age of 75 (Alzheimer’s Association, 2022). It is reported that, an estimated 8 million AD-patients are present worldwide (Alzheimer’s Association, 2022) and 115.4 million, AD-patients might be added by 2050, a threefold increase. People over 65 have a prevalence of 6%, people over 80 have a prevalence of 20%, and people over 95 have a prevalence of almost 95%. Adults patients die from AD as the sixth greatest cause of death (Alzheimer’s Association, 2022). A total of 7–9 years is the typical time span between the beginning of symptoms and the patient’s death.

**FIGURE 1:**
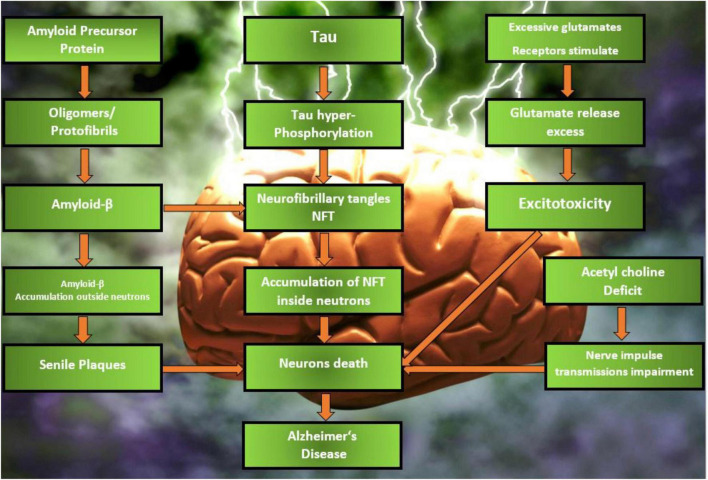
Different phases of pathophysiology of AD.

Neuronal and synapses are compromised in AD, and neurofibrillary tangles, senile plaques, and neurodegeneration are pathological hallmarks (Wilson, 2011). Tau proteins are hyperphosphorylated, resulting in neurofibrillary tangles (also known as tau tangles), which are paired helical filaments. Axons are the most common location for tau proteins. Hyperphosphorylation of these proteins is the result of cellular dysregulation. Neurofibrillary tangles have a devastating effect on axonal integrity and the transport of neurotransmitters (Sun et al., 2003). As the Aβ protein accumulates on the exterior of neurons, it forms senile plaques. Cleavage of Aβ precursor protein (APP) through proteolytic cleavage results in the production of Aβ. The APP, which is found in the brain, is involved in neurogenesis, synaptic formation, cell signaling, axonal transport, and plasticity. When secretases, such as β-secretase and γ-secretase, are used to release Aβ peptides with 40 or 42 amino acids from APP, Aβ oligomers and finally Aβ plaques are formed, and these plaques are then processed in further ways. Deposition of Aβ plaques and neurofibrillary tangles causes nerve cell deterioration and death. The pathophysiology of AD has been described in Figure 1.

Cholinergic neurotransmission is essential for a variety of cognitive functions, including memory, learning, attention, sleep, and sensory perception, as well as the stress response. Deficits in acetylcholine (ACh), a neurotransmitter critical to cognitive function, have been linked to AD (Francis, 2005). A deficiency in cholinergic transmission may have a significant impact on both cognition and behavior, so be aware of this possibility (Hampel et al., 2018; DeTure and Dickson, 2019). When cortex is disrupted, cholinergic input also gets affected. CA3 cholinergic receptors have been shown to affect information and memory encoding (Rogers and Kesner, 2004). Brain activity in Alzheimer’s sufferers was found to be lower than normal. Because of this, the cholinergic theory acquired a lot of support. Synaptic transmission and plasticity are greatly influenced by NMDA (N-methyl-D-aspartate receptor) receptors (Liu et al., 2019; Lituma et al., 2021). It seems that they help neurons survive by activating a neuronal survival pathway (Hardingham et al., 2002). Neuronal death and degeneration have been linked to NMDA receptor blockade (Ikonomidou et al., 1999). Synaptic NMDA receptor signaling has a detrimental effect on the survival of neurons when it is insufficiently expressed. Neuronal injury and neuronal death result from over activation of glutamatergic signaling (Rothman and Olney, 1986). Neuronal cell loss begins in memory and learning-related regions of the brain and extends throughout the body (Brunholz et al., 2012). Genetic mutations are responsible for a small number of cases of AD. There are mutations in the APP and PS1 and PS2 protein genes (presenilin 1 and 2) (Li et al., 2016; Duncan et al., 2018; Dehury et al., 2019). APP and presenilin gene mutations are seen in familial AD, i.e., PS1 and PS2 are present, but not tau. Several variables have a role in the development of the illness, and age is one of them. The average age of patient is 65 years old when they expected the first diagnosed. AD is more likely to develop in those with a family history of the disease, as do those who have the apolipoprotein E allele 4 (König and Stögmann, 2021). The primary aim of review is to present the readers the current treatment and diagnosing strategies for AD for more targeted approach.

## Methodology

The literature review was conducted using various search engines such as PubMed,^1^ Science direct,^2^ Scopus,^3^ Google scholar.^4^ The studies from the years 2010–2022 were chosen with key terms such as “Alzheimer’s disease,”, “neurological disorders,” “amyloid,” “nanoparticles,” “polymeric micelles,” “dendrimers,” “nanotechnology,” and “liposomes”. The papers which were indexed in the above-mentioned search engines were only.

## Why Nanotechnology Is Important for the Research of Alzheimer’s Disease

The neuronal “milieu” is protected from external chemicals by the blood–brain barrier (BBB), ensuring chemical balance in neuronal circuits and synaptic transmission. The primary contact between the blood and the brain is established by the endothelial cells of the cerebral capillaries, which constitute this barrier. The BBB is the most significant impediment to the development of novel CNS therapeutics and biologics. Pharmaceuticals, including most tiny compounds, do not typically enter or exit the BBB (Cavalu et al., 2020a). Numerous efforts have been made over the last decade to address this critical issue by devising various techniques to facilitate medication transport over the BBB. In recent years, nanotechnology-based approaches have grown in prominence due to their ability to circumvent the restrictions imposed by BBB crossing. Controlled medication delivery and release for a variety of CNS diseases may be achieved using a variety of lipidic, polymeric, inorganic, and other kinds of nanoparticles (NPs) (Cavalu et al., 2020b).

As a result, NP-based treatments for AD have mostly focused on preventing Aβ from aggregating or sequestering the peptide in order to reduce its brain level (the so-called “sink effect”) (Matsuoka et al., 2003). Sink-effect-capable nanoliposomes (NL) with phosphatidic acid or cardiolipin were developed by Gobbi et al. (2010). Studying these NL *in vitro*, researchers found that they were highly sensitive to Aβ and had its protective effect (Bereczki et al., 2011). The findings of Mourtas et al. (2011) and Canovi et al. (2011) show that NL coated with an anti-A monoclonal antibody, which has strong affinity for Aβ either *in vitro* or *ex vivo* on post-mortem AD brain tissues, may suppress Aβ aggregation *in vitro* and *in vivo* (Taylor et al., 2011). The capacity of fullerenes C60 to prevent Aβ fibrillization is being studied in a similar fashion (Podolski et al., 2007).

By shielding neurons from oxidative damage, NPs may alleviate the symptoms of AD. They may be used as anti-oxidative agents, but they have toxic effects that are especially dangerous for those with liver and brain impairment. Several studies have shown that NP may be used to address these issues (Liu et al., 2009b). The iron chelator 2-methyl-N-(2’-aminoethyl)-3-hydroxyl-4 pyridinone was found to be able to protect neurons from Aβ toxicity and prevent Aβ aggregation *in vitro*, without affecting cell growth or proliferation, as demonstrated by Liu et al. (2009a).

## Drug Loaded Nanomedicines for Alzheimer’s Disease

### Liposomes

When it comes to delivering medication over the BBB, the phospholipid bilayer of liposomes is the most likely answer. Nonetheless, the BBB is not permitted to be crossed. In order to increase liposomal carrier transport across the BBB, many surface changes have (Spuch and Navarro, 2011). The BBB’s surface may include several proteins, peptide, antibody, and other ligand receptors. The use of surface-active ligands, such as those present in these compounds, facilitates transcytosis. Cationic liposome absorption into the BBB is also occurring at the same time as transcytosis. To facilitate their passage through the body, liposomes are coated with nutrients such as glucose (Noble et al., 2014). Once in the brain, the liposomes begin the process of passive diffusion, which is activated by the brain’s own passive efflux (Noble et al., 2014). The rate at which the chemical is released is unaffected. When the patient’s physiological conditions change, fresh techniques have been created that adapt to these changes and manage the drug’s release accordingly. Liposome drug release may be triggered by pH variations, enzyme activity, or changes in glutathione levels, to name just (Cavalu et al., 2002; Andresen et al., 2005; Malam et al., 2009). Amyloid peptides interact with ACh, causing amyloid plaque to disintegrate and reducing inflammation in the brain. This helps to preserve healthy neurons as well as treat AD once it has been released.

Liposomes can be modified using ligands for the internalization of active, as BBB have negative charge and electrostatic interactions takes place (Joshi et al., 2014, 2015). PEG11 or polysaccharides may be added to the surface of liposomes to enhance their pharmacokinetic profile, enabling them to remain in circulation for longer periods of time and increasing their distribution into the brain (by inhibiting their rapid clearance *via* the RES12). Despite the fact that “stealth” liposomes were able to significantly shorten the time it takes for liposomes to circulate, there is no guarantee that liposomes will cross the BBB. There are a number of recent approaches for “stealth” liposomes to add extra features, such as physiologically active ligands such as peptides, antibodies or small compounds that bind directly to receptors or target transporters overexpressed on brain endothelial cells. As a result of the specificity of receptor and ligand interactions, receptor-mediated transcytosis has been identified as the most successful and often used method for liposome delivery to the brain. The most often targeted receptor is TfR13, a transmembrane glycoprotein found in abundance in brain endothelial cells. There have been studies using tf14-functionalized liposomes for BBB targeting, however, endogenous Tf prevents them from binding to the receptor (Chen et al., 2014), therefore antibodies against TfR (which binds to the receptors at discrete sites) have been used to eliminate ligand competition (Markoutsa et al., 2012; Mourtas et al., 2014). Another iron-binding glycoprotein found in mammals, lactoferrin, which binds to Lf15 receptors and is found in abundance on the BBB, has also been used to functionalize liposomes. Using receptor-mediated transcytosis, lactoferrin-modified liposomes have been generated as functionalized nanocarriers for BBB crossing (Chen et al., 2010).

Curcumin and two BBB piercing peptides were combined with the preceding research to determine whether a synergistic effect could be achieved by combining the two BBB penetrating peptides, and to test if curcumin interfered with the functioning of the BBB targeting ligands (Papadia et al., 2017b). Curcumin derivative and two BBB binding ligands are added to the surface of multifunctional liposomes (targeting transferrin and LDL receptors). For Aβ inhibition, the liposomes were tested in hCMEC/D3 cells with surface-modified liposomes. These surface-modified liposomes were analyzed for the influence of one or more alterations on the BBB targeting and amyloid binding properties of these liposomes. Studies on FVB and APP/PS1 transgenic mice also shown that curcumin derivatives did not interfere with BBB specific ligands’ ability to function (Papadia et al., 2017a,b). An AD nanomedicine made of PEGylated immuno liposomes and two monoclonal antibodies (MAbs) targeting the transferrin receptor and Aβ peptide was developed. Streptavidin-biotin complex and maleimide conjugation were used to affix OX26, an anti-transferrin receptor mAb, and 19B8 to the liposome surface. After being administered intravenously to male Wistar rats, these immunoliposomes were taken up by porcine brain capillary endothelial cells and successfully permeated the BBB *in vivo*. The extracellular trafficking of 19B8 across the BBB was made possible by the disease’s persistently leaky BBB. A potential technique for AD treatments was found to be the use of immunoliposomes with two ligand-targeting antibodies (Loureiro et al., 2015).

Rivastigmine and CPP-loaded liposomes may be used to treat AD, according to Yang et al. (2013) experiment. It exhibited cognitive and behavioral development, had fewer side effects, and increased the concentration of medication in the brain, resulting in great therapeutic efficacy in this treatment modality. Beta sheet blocker peptide may be added to liposome vehicles to prevent Aβ protein buildup in the brains of AD patients (Birks and Harvey, 2018). If NPs are supplied intranasally, researchers say that all of the liposomes encapsulated with rivastigmine showed effective drug transport to the brain and high BBB penetration. Nanoformulations of monoclonal antibodies have shown promising outcomes in the treatment of AD. Research with monoclonal antibodies tagged with curcumin showed that they were able to attach strongly to the neurotic plaques in the brain tissue of AD patients and prevent the formation of Aβ peptide. It was shown that curcumin-loaded liposomes were capable of discoloring the accumulated Aβ in mice (Qizilbash et al., 1998). It was shown that the breakdown of Ach by the enzyme Acetyl choline esterase (AChE) could not occur in the presence of rivastigmine-loaded liposomes in solution with sodium taurocholate, according to another study on these liposomes. To improve AChE inhibitor effectiveness, folic acid may be supplied in liposome form through intranasal route. Because the reticuloendothelial system is protected by PEG-coated liposomes, they are very successful in the treatment of AD. To that end, glutathione PEGylated liposomes have been shown to improve medication absorption across the BBB. Infusion of rivastigmine-tagged liposomes and rivastigmine solution into an artificial AD model treated with aluminum chloride indicated the improvement in deteriorated memory that was encouraged by aluminum chloride.

Glutathione (GSH) and ApoE were both transported to the AD-specific location when treated with active ligands by Kuo et al. (2021). It has been shown that increasing the Stearic acid (SA) percentage in liposomes while reducing the PC proportion affected the entrapment efficiency of drugs and the stability of liposomes overall. By enhancing the BBB permeability of medicines, GSH-ApoE-PC-liposomes boosted the brain-targeting capacity. ApoE-coated liposomes improved endocytosis of Curcumin (CURC), Quercitin (QU), epigallocatechin gallate(EGCG), rosmarinic acid(RA), and phosphatidylcholine(PC) into SK-N-MC cells *via* engulfing with low-density lipoprotein receptor (LDLR), as shown in this study. To identify A, liposome-encapsulated PC had to rely on a binding affinity that was very particular. Anti-apoptotic effects of GSH, ApoE, CuRC, EGCG, and PC liposomes were shown by immunofluorescence labeling and western blot analysis in Aβ_1–42_ infected cells following treatment with GSH-ApoE–CURC–QU–EGCG-RA-PC liposomes. The GSH-ApoE-PC-liposome triple targeting formulation was used to penetrate the BBB and deliver CURC, QU, EGCG, and RA to the AD-specific location concurrently. A multifunctional GSH-ApoE-CURC-QU-EGCG-RA-PC-liposomes method for AD control was discovered in this study.

Researchers developed non-invasive method of analysis of AD based on liposomes (Monteiro et al., 2022). An Aβ40 peptide liposome encapsulated in dipalmitoyl phosphatidyl glycerol (DPPG) liposomes and attached to a polyethylene imine-coated screen-printed carbon electrode is used to detect autoantibodies against Aβ40, a possible biomarker in plasma samples. Immunosensing utilizing a 2-bilayer PEI/DPPG + anti-Aβ40 autoantibody was able to distinguish plasma and CSF from plasma and CSF samples from three patients with AD by applying currents at 0.45 V. In addition, the CSF voltammograms were able to distinguish healthy samples from those of patients with higher performance. Hence, the ability to identify AD in plasma samples from patients with a confirmed diagnosis, which definitely has larger notoriety, was a first step toward the primary goal: a successful diagnosis of AD in the preclinical stage and plasma. The comparison of peripheral blood and CSF in diagnostic techniques is also critical. Some other studies citing the use of liposomes has been listed in Table 1.

**TABLE 1:** Latest researches citing use of liposomes for treatment of AD.

Active drug	Outcome	References
Icariin and Tanshinone IIA	The pharmacodynamic analysis *in vivo* demonstrated that Ang2-ICA/TSIIA liposomes could improve AD-like pathological features in APP/PS1 mice, including inhibiting neuroinflammation and oxidative stress, reducing apoptosis, protecting neurons, and improving cognitive function	Monteiro et al., 2022
Hydroxy-α-Sanshool	Liposomes were not significantly toxic to the nasal mucosa and effectively alleviated D-galactose-induced learning memory deficits and protected mouse hippocampal neuronal cells	Li et al., 2022
Imatinib Mesylate	The liposomes effectively improved the brain deposition of drug in brain from formulation compared to pure drug solution as indicated by AUC from *in vivo* experiments	Saka et al., 2021
Transferrin-Pep63	Pep63 effectively inhibited the binding between EphB2 and Aβ oligomers after release from liposomes and rescued NMDA receptors trafficking, the basis of synaptic plasticity	Yang et al., 2021
Rosemary extract	All optimal Nano-Liposomes samples showed statistically significant higher antioxidant capacity (>94.15%) compared to Rosemary extract (90.04%)	Shalabalija et al., 2021

### Nanoparticles

#### Chitosan Nanoparticles

Chitosan (CH) is a naturally derived cationic polysaccharide composed of glucosamine and N-acetylglucosamine copolymers (Chen et al., 2010). It is a deacetylated chitin found in lobster, crab, and shrimp shells. The enzyme lysozyme hydrolyzes chitosan into non-carcinogenic, non-toxic, and non-immunogenetic amine sugar compounds that may be absorbed and destroyed by the human body. Chitosan is self-medicating, lowering cholesterol, and promoting ulcer healing and wound healing (Chopra et al., 2020, 2021b,2022a,b,c; Singh Bakshi et al., 2022). Chitosan NPs are smaller than 70 nm, resulting in a greater surface-to-volume ratio (Cavalu et al., 2018). Chitosan’s drug encapsulation efficiency rises with decreasing molecular weight (Xu and Du, 2003; Yang and Hon, 2009). However, research shows that chitosan breakdown rises with molecular weight decrease (Szymańska and Winnicka, 2015).

SpBMP-9, a peptide generated from bone morphogenetic protein-9 (BMP-9), has recently been delivered to the central nervous system (CNS) *via* an alginate-Chitosan NP-based method (Lauzon et al., 2018). Peptide SpBMP-9 (derived from the growth factor BMP-9) is a short peptide that has been shown to stimulate cholinergic neuron development and inhibit GSK3a (a kinase of tau protein) (Beauvais et al., 2016). Peptide-loaded Alg/CS-based NPs enhanced the viability of SH-SY5Y cells when compared to controls. Higher neurite outgrowth and enhanced expression of neuronal markers (such as vesicular acetylcholine transporter (VAchT) and neuron specific enolase (NSE)) were evidence that released SpBMP-9 from NPs stimulated SH-SY5Y cells’ differentiation into adult neurons.

Elnaggar et al. (2015) synthesized piperine-loaded chitosan NPs in another investigation. After loading with piperine, the chitosan NPs produced in this work had an average diameter of 230 nm. Dialysis (for 2 h) of piperine-loaded chitosan NPs indicated that the controlled release of piperine was greater than that of unbound piperine in the *in vitro* investigation. Using an animal model of senile dementia of the Alzheimer’s type (SDAT), researchers have also undertaken *in vivo* experiments on the cognitive impairments caused by colchicine. Increased AChE secretion in AD brains may be attributed to the increased production of free radicals by colchicine. Cognitive impairment is exacerbated by an increased amount of AChE in the brain, which lowers brain Ach concentrations. Thus, Elnaggar et al. (2015) concluded that piperine-loaded chitosan NPs might be a suitable option to improve cognitive impairment in the brains of patients with AD based on *in vivo* experiments.

Chitosan NPs have been shown to be effective in the intranasal administration of FDA-approved AD medications like Rivastigmine in multiple studies. Research by Fazil et al. (2012) examined the bioavailability of Rivastigmine into the brains of Wistar rats. Diffusion-based mechanisms were found *in vitro*, however *in vivo* experiments demonstrated that the drug was better retained in brain regions when administered as a solution rather than pure. Tween-80 coating of chitosan NPs with Rivastigmine was shown to be more effective in reversing amnesia in Swiss albino mice than chitosan NPs without coating by Nagpal et al. (2013). In contrast to the rapid biphasic release seen with Rivastigmine alone, adding coated or uncoated NPs produced a more gradual biphasic release. As an alternative, trimethyl chitosan (TMC) covalently attached to surface-modified polylactide (PLGA) NPs has been shown to transport Coenzyme Q10 to APP/PS1 transgenic mice (Wang et al., 2010). Memory was much improved, and it was hypothesized that the NP system was able to penetrate the BBB through adsorption-mediated transcytosis and that it was less hazardous.

In spite of the promise of Growth Factors (GF) for treating AD, only a few studies have been undertaken on their entrance into the brain, and most of the studies that have been published have employed chitosan solutions rather than NPs in their research. Nanocapsules of chitosan-based nanocapsules, such as BMP-2 and bFGF, may effectively encapsulate and release GFs like these (Lai et al., 2013). Sprague Dawley rats’ brains absorbed BDNF in a chitosan solution (0.25 percent w/v) 13 times quicker than did BDNF alone, as shown by Vaka et al. (2012). A spray solution containing the chitosan chitin was shown to be more effective than intravenous injections in terms of brain/serum concentration ratio and acetyltransferase activity, as well as reducing memory impairment (Feng et al., 2012). Because of this, chitosan seems to have the potential to be an ideal carrier for therapeutic GFs across the BBB. To further understand how NPs aid in the delivery of GF, more research is required.

The chitosan NPs’ surface charge is also an important factor that might influence their therapeutic efficacy. The presence of amine groups on the C6 position of the pyranose ring in chitosan gives it a cationic character, which may be favorable or harmful in biomedical applications. Using this positive charge, chitosan NPs may be easily synthesized, for example by self-assembly or ionotropic gelation (Jiang et al., 2018). The negatively charged sialic acid moiety of the mucosa is electrostatically bound by the hydrophilic and cationic amino groups, which aid the polymer’s mucoadhesivity. Additional evidence suggests that the negative charge of chitosan NPs may play a role in both their cellular absorption and their dispersibility (Jiang et al., 2018). It is possible that the finding that PEG-modified chitosan NPs in MTT tests were less hazardous to cells than uncoated chitosan NPs may suggest that excessive positive charge attributable to free amine groups might affect the integrity of membranes to the point of causing cell death (Malhotra et al., 2013). Chitosan’s cationic nature may potentially impair the encapsulation effectiveness of cationic drugs. Since the degree of dispersion of individual particles is significantly influenced by electrostatic repulsion, surface charge on chitosan-based drug nanocarriers is also critical for their stability. The mild electrostatic repulsive interactions between individual particles explain why NPs with negative surface charge display poor aggregation stability (Ormanci et al., 2014). It is because of this that the RES macrophages can rapidly aggregate and remove these particles from the bloodstream. API nanocarriers with high positive surface charges, on the other hand, are unfavorable because they are more easily phagocytized than NPs with lower positive charges (He et al., 2010) and because they were demonstrated to have an acute toxic impact on the BBB right away. Therefore, nanoparticles for the treatment of AD must have a tiny positive surface charge in order to enhance blood circulation and reduce BBB toxicity (Ormanci et al., 2014). Finally, the surface charge of chitosan NPs has a significant influence on Aβ aggregation (Zhang and Neau, 2001; Chang et al., 2018), however the precise impact of positive and negative charges has been disputed (Cabaleiro-Lago et al., 2010; Assarsson et al., 2014a; Liu et al., 2015b). Although positive NPs have been shown to prevent Aβ fibrillogenesis in a few investigations, their Aβ aggregation-stimulating impact has been observed in others (Luo et al., 2013; Assarsson et al., 2014b). The suppression of Xu et al. (2009), Chan et al. (2012) as well as the promotion of Elbassal et al. (2016), Kim et al. (2016) of Aβ fibrillogenesis have both been linked to negative charges. Most likely the NPs’ ability to prevent or promote amyloid-beta aggregation relies on a variety of parameters, including surface charge, hydrophobicity and pH, and the kind of Aβ species.

#### PLGA Based Nanoparticles

**FIGURE 2:**
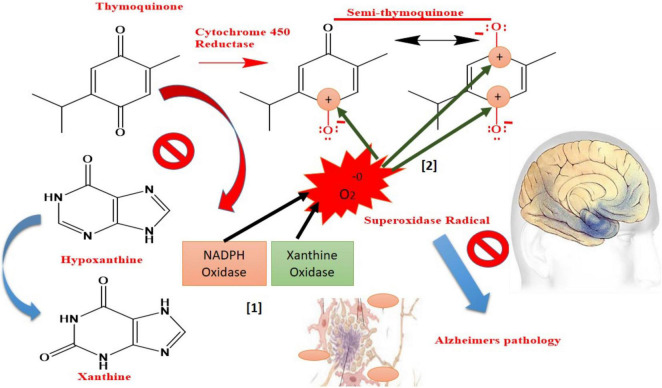
Mechanism of “Thymoquinone” drug toward oxidative stress-mediated AD symptoms.

*Nigella sativa* seed essential oil has been shown to have a bioactive ingredient known as Thymoquinone (TQ), which has been shown to have a wide range of medicinal applications (Javidi et al., 2016). Several early pharmacological studies have been conducted to investigate the therapeutic usage of TQ and additional study is needed to determine its usefulness in neurological illnesses. TQ has recently been shown to have high promise in the treatment of AD by Abulfadl et al. (2018). To achieve any meaningful therapeutic effect, TQ’s short bioavailability and rapid elimination hinder its potential to be developed in any traditional dosage form due to its high lipophilicity and strong plasma protein binding (99%) (Alkharfy et al., 2015). The targeted drug delivery system (TDDS) is effective, but the BBB and other cerebrospinal barriers limit the spread of any foreign particles between the CNS and blood. However, these protective barriers are extremely important for normal CNS physiology. Reducing medication delivery systems’ capacity to treat brain illnesses may be hindered because to several limitations. In order to transport drugs over the BBB without diminishing their potency, TDDS is needed (Pires et al., 2009). Polysorbate-80 (P-80) coated NPs containing TQ might be a viable and reliable technique of delivering nanoscale delivery to the brain over the BBB (Yusuf et al., 2021). When hydrolyzed into its harmless endogenous metabolites, such as glycolic acid and lactic acid, Poly(lactic-co-glycolic acid) (PLGA) is a biodegradable polymer (Kumari et al., 2010). However, while PLGA is a commonly utilized polymer for CNS specific drug administration, its hydrophobic nature tends to opsonize and remove it *via* the reticulo-endothelial system (RES) (Sempf et al., 2013). Surfactant P-80, a non-toxic, non-ionic, biodegradable, and hydrophilic surfactant, is ideally suited to achieve this job because its coating protects PLGA NPs from being opsonized and eliminated (Kreuter et al., 2002). P-80 inhibits P-glycoprotein efflux by adsorbing apolipoproteins to the surface of the cell and mimicking LDL to pass the BBB through receptor-mediated endocytosis. TQ release from the PLGA matrix may be explained by a variety of processes, including matrix degradation and drug diffusion (Fredenberg et al., 2011; Kamaly et al., 2016). The autocatalytic hydrolytic breakdown of PLGA to lactic and glycolic acid is a major support for drug release from matrix through diffusion via pore creation (diffusion). High porosity and strong drug diffusion may be achieved by autocatalytic breakdown of the matrix. TQ from P-80-TQN was readily released due to P-80 coating’s hydrophilicity (Tıʇlı Aydın et al., 2016). An initial quick burst lasted for two hours, followed by a persistent release for P-80-TQN’s biphasic release paradigm. One possibility is that the early burst of TQ is a consequence of TQ restricted to the outer surface. Due of the PLGA matrix swelling and disintegration, it may have been responsible for the early burst of TQ (Zhang and Feng, 2006; Tıʇlı Aydın et al., 2016). TQ and PLGA interaction may have resulted in further sluggish release, which may have limited the release of TQ. The TQ acts primarily by inhibiting enzyme Xanthine-oxidase and reducing the production of superoxide radicals, while semi-TQ produced by cytochrome 450 reductase provides electron deficient platforms at 1 and 4th positions, providing electron deficient centers for superoxide radicals (As shown in Figure 2). Both processes have the potential to lower the amount of superoxide radicals produced as a result of decreased OS and AD.

As an anti-oxidant and anti-inflammatory, curcumin has been studied as a possible biological treatment for a variety of health concerns (Chopra et al., 2021a), including cancer, sepsis, heart, and brain illnesses, including AD (Yallapu et al., 2010, 2012; Chopra et al., 2016). During human clinical studies, cur substance was shown to be very safe, with no serious side effects at the higher dosage (4 g/day). Intermolecular hydrogen bonds may also be used to interact with Aβ and iron in plaques without the need of additional chemical linkers. The non-polar regions of Aβ plaques form hydrophobic interactions with the symmetrical phenyl and methoxyl groups of cur molecular chains, allowing them to attach readily to Aβ plaques. Cur’s hydroxyl and diketone groups connect to the polar regions of Aβ plaques by hydrogen bonding, further stabilizing the bonds. Researchers prepared, curcumin and Se NPs loaded PLGA nanospheres for targeting AD (Huo et al., 2019).Neuroimaging of Alzheimer’s-treated mice was used to assess the nanospheres’ capacity to recognize Aβ plaque. These transgenic mice (5XFAD) were utilized in this study because of their ability to display plaque, which increased significantly in quantity as the animal became older, fluorescence intensity was minimal in both Cur and Cur/PLGA groups. In contrast, the Se/Cur-PLGA nanospheres show an increased fluorescence intensity, which confirms that Se NPs on the nanospheres surface helped to improve BBB penetration. Astonishingly, Se/Cur-PLGA was spread throughout the brain slice of the mice, compared to Cur-PLGA. When the Se/Cur-PLGA nanospheres were found mostly on the plaques, it can be directly shown that these nanospheres were attached to Aβ plaques by traversing the BBB in its entirety. Additional neuroprotective features of Cur compounds include their high levels of antioxidant and anti-inflammatory activity as well as their ability to neutralize amyloid and tau hyperphosphorylation. It is possible for curcumin molecules to interact with Aβ oligomers *via* hydrophobic contacts in the non-polar regions of Aβ oligomers and the hydrophobic chains of curcumin. Hydrogen interaction between the polar regions of Aβ oligomers and curcumin’s hydroxyl groups may further solidify these hydrophobic connections. Aβ oligomer toxicity in AD may be reversed by curcumin molecules, which act as an obstructive agent.

Dhas and Mehta (2021) prepared flavonoid, i.e., Curcumin based PLGA NPs for AD for reducing oxidative stress. Cur’s low solubility, rapid metabolism, and bioavailability have limited its use in medicine because of these three things. Not only does Cur have a low solubility, but it has a low solubility at different pH ranges, like 11 ng/ml in water at pH 5, 0.0004 mg/ml in water at pH 7.4. Under normal conditions, Cur breaks down quickly. The main product is trans-6-(4-hydroxy-3-methoxyphenyl)-24-dioxo-5-hexenal, which is also broken down into ferulic acid, feruloyl methane, and vanillin. Cur is also prone to photodegradation, which could make it difficult to store for a long time. Due of its biocompatibility, biodegradability, and non-toxicity, PLGA was chosen as the study’s main material. It can readily be functionalized across the surface with other materials and has a high drug loading capacity (Khan et al., 2018; Mehta et al., 2019). Chitosan (CH), a cationic mucoadhesive polymer, was also included in the research since it has the potential to produce gels by absorbing water, which will aid in prolonging the duration of action at the site of action. According to several reports, CH aids in the opening of tight junctions and improves medication penetration through the nasal mucosa when administered orally (Islam et al., 2015). It is therefore possible to use CH to enhance the residence duration and improve medication permeability by using this material as a shell material.

Therapeutic and biological uses of AuNPs have garnered significant interest. For the therapy of neurodegenerative illnesses, AuNPs have been identified as a viable option. Aβ aggregation is inhibited, and Aβ fibrils are dissociated, which has a synergistic impact on suppressing and dissociating Aβ faults. Their distinctive optical characteristics, chemical stability, electrical conductivity, biocompatibility, and catalytic activity are just some of their many other impressive attributes. Both their surface area and their protein-binding adsorption capability are huge. They may be used to identify Aβproteins by combining with antibodies. Increased signal and improved electron transfer efficiency are also a result of their incorporation (Elbassal et al., 2017; Muller et al., 2017; Song et al., 2018; Cao et al., 2019; Qiao et al., 2021). Consequently, they have been frequently used in the development of AD biomarker biosensors (Meenambal and Srinivas Bharath, 2020). AuNPs-based AD diagnostic techniques would benefit greatly from these exceptional qualities and traits.

A number of trials have been done to find an effective way to prevent the aggregation and fibrillation of Aβ in AD. One of AD’s most striking pathological features is the accumulation of A peptides into insoluble amyloid fibrils (Pradhan et al., 2018). AD may benefit from the use of Aβ aggregation inhibitors. The A fibrils may be destabilized *in vitro* by a variety of drugs, which prevent Aβ aggregation and neurotoxicity. The inhibitory effects of NPs have been extensively explored. Protein aggregation may be effectively inhibited by the functional NPs. Preformed fibrils may be disintegrated by light-activated AuNPs containing peptides. When the nanocarrier is properly surface functionalized, harmful ions are prevented from being released from the nanocarrier (Meenambal and Srinivas Bharath, 2020). NPs may break tiny fibers and prevent them from aggregating. By slowing down the nucleation process, small AuNPs may prevent Aβ aggregation and fibrillation in experiments. As a result, the synthesis of AuNPs may give theoretical insights for therapeutic candidates to treat AD.

An amyloid fibril formation inhibitory effect is shown for gold nanoparticles (AuNP) coated with metal-phenolic networks (MPN). Studies have shown that MPN may work synergistically to prevent amyloid aggregation (Zhang et al., 2019). Metal ions accelerate Aβ aggregation and promote the creation of neurotoxic reactive oxygen species, which lead to the beginning of AD. Despite the fact that metal chelators may block these effects, most of these chelators cannot traverse the BBB or distinguish metal ions associated with toxic Aβ plaques from those in normal biological systems. They can only reduce these effects. AuNPs may be used in the biomedical area because of their high BBB permeability, anti-Aβ aggregation, and great biocompatibility. Molecular interactions have a strong influence on the production of amyloid in the human body. In biological systems, the interfaces are often not flat and exhibit a wide range of sizes and shapes. Because of the enrichment impact toward Aβ, they have the ability to significantly speed up fibrillation. Binding sites are also increased. The fibrillation kinetics may be further altered by interactions with Aβ peptides at these locations. Gao et al. found that large AuNPs accelerated Aβ fibrillation, but smaller ones slowed it down (Gao et al., 2017). Peptide chains were reduced in aggregates generated by AuNPs, and the smaller NPs showed the highest inhibitory effectiveness (Hou et al., 2020). The findings suggested that particle size may have a significant impact on protein folding when adsorbed on NPs.

It is also vital to consider the form of AuNPs in the transport of drugs, bio-labeling and internalization. Gold nanospheres (AuNSs) and gold nanocubes (AuCubes) were created through seed-mediated development of AuNPs by Wang et al. (2019) (AuNCs). Although their surface structures differed, their surface chemistry was quite similar (Wang et al., 2019). Two AuNPs were used to study the secondary structure and fibrillation kinetics of Aβ (1–40) using different microscopic and spectroscopic methods. It is also possible to change the form of NPs to monitor the flow rate, cell absorption, and systemic dispersion of the drug-loaded nanocarrier in the body (Meenambal and Srinivas Bharath, 2020).

The secondary structure of the fibrils generated by AuNPs is influenced by their shape. Peptide secondary structure may be studied using the FTIR. An amyloid fibril’s sheet structure, in which the chain folds perpendicular to the long axis of the fibrils, is prevalent. It has been shown that AuNPs may increase sheet content and lead to the development of amyloid fibrils (Wang et al., 2019). It was found that the size, surface charge, concentration, and shape of AuNPs had an influence on the integrity of BBB. AuNPs are attached to Aβ structures in a selective manner. Although these AuNPs conjugates have been widely exploited as photothermal absorbers for putative AD therapy, their overall negative charge could prevent them from passing across the BBB. The integrity of the BBB was severely compromised by the presence of small AuNPs (Ruff et al., 2017). AuNPs, a nanotechnology-based brain delivery system, may hold the key to better brain treatment (Teleanu et al., 2018).

#### Antibody Decorated Nanoparticles

Adverse events like as meningoencephalitis may occur as a result of administering immunotherapy dosages to treat AD (Moretto et al., 2007; Hoskin et al., 2019). The best option to using immunotherapy to identify and dissolve protein aggregates in brain cells is to use NPs coated with antibodies for particular target proteins. Antibodies coated with metal oxide NPs are used in secondary ion mass spectrometry to image proteins related with AD in the brain (Moon et al., 2020). Amyloid-bearing cells in AD have been targeted using nano-vehicles coated with chitosan and Aβ fragments. Contrast chemicals such as FITC and Alexa Fluor are used to enhance NP-Aβ absorption across the BBB (Agyare et al., 2008). Class A receptor activator XD4 (W20/XD4-SPIONs) and A oligomer-specific scFv-AbW20 coupled to superparamagnetic iron oxide NPs (SPIONs) show promising outcomes in the treatment of AD (Liu et al., 2020a). Superparamagnetic iron oxide NPs linked with A-oligomer-specific antibody and class A scavenger receptor activator reveal excellent early diagnostic potential for AD (Liu et al., 2020b).

#### Magnetic Nanoparticles

Typically, gadolinium-based contrast agents are accessible as biocompatible chelates of Gd3 +, which prevent any competitive inhibition of biological processes that involve calcium ions from occurring during the procedure. Despite the fact that various Gd-based contrast agents are commercially available, their clinical use has been discontinued owing to the danger of nephrogenic systemic fibrosis as a result of the buildup of Gd in the tissues of patients. IONPs are considered to be a safer alternative to gadolinium in MRI contrast agents. Because the IONPs are more effective than gadolinium, they may be used at lower concentrations to achieve the same relaxation changes in the surrounding water molecules. In part because of their smaller size, they exhibit superparamagnetism, with each molecule serving as a separate magnetic domain, and as a result, they get magnetized to a higher degree when compared to the same number of Gd-DTPA NPs, which are routinely utilized as T1 contrast agents (Blasiak et al., 2013). In order to achieve certain qualities such as biocompatibility and solubility, IONPs may be functionalized with desired motif sequences. They are able to target brain areas that would otherwise be inaccessible due to their tiny size when paired with their small size. However, although the nephrotoxicity of gadolinium NPs is a source of worry, it has been hypothesized that the IONPs get absorbed into the body’s iron stores after a few days in the system (Daldrup-Link, 2017). In this way, they may be used in longitudinal investigations of follow-up detection tools after therapeutic therapies. The FDA-approved IONPs have core sizes ranging from 50 to 200 nm, good biodistribution, and biocompatibility, and many of them were approved for clinical use, including Resovist^®^, Clariscan^®^, and Feridex^®^, before being removed from the market (Cardona et al., 2016). They are traditionally synthesized by co-precipitating a mix of Fe^2+^ and Fe^3+^ salts such as FeCl_2_.4H_2_O and FeCl_3_.6H_2_. The NPs precipitate and are magnetically collected, after which they are dried under high pressure and temperature in a vacuum (Mohammadi and Barikani, 2014). Additional factors influencing the selection of appropriate IONPs include their bioconjugation, decreased size, stability, solubility, and biocompatibility when exposed to physiological circumstances, to mention a few. They may be endowed with specialized capabilities by selectively attaching to functional patterns found in antibodies, carbohydrates, proteins, and tiny biomolecules, among other substances. Fe^3+^ ions have a larger r1 relaxivity than other ions, indicating that IONPs have the potential to be developed as both T1 and T2 contrast agents. After MRI, the existence of IONPs in the tissue is often verified using histological staining, such as Perl Blue, in the majority of pre-clinical research investigations.

In order to see amyloid structures in the sick brain, anti- Aβ antibodies have been conjugated to IONPs. Intranasal injection of NU-4 antibodies, which are designed to precisely target harmful soluble A oligomers, was reported to bind (24–48)-unit Aβ oligomers in the brains of AD animals after intranasal delivery (Viola et al., 2015). These antibodies were also discovered utilizing magnetic resonance imaging (MRI) when probed on probable AD human brain slices and a 5xFAD mouse model. IONPs that were dual functionalized with the amyloid-binding dye Congo Red and the antioxidant Rutin were shown to aggregate with Aβ plaques in the brains of AD transgenic mice and to relieve memory impairments (Hu et al., 1994). In order to detect aggregation in the sick brain, IONPs were conjugated to A(1–42) peptides, which were chosen because of their natural affinity for Aβ plaques. Following intravenous injections of these probes into the transgenic mice’s brains, MRI images and immunolabeling investigations revealed that they were present in AD-associated brain areas such as the cortex and hippocampus (Wadghiri et al., 2013). Other characteristics of AD may be detected and diagnosed using magnetic NPs. For example, under the influence of alternating magnetic fields, IONPs functionalized with anti-tau antibodies may identify tau proteins in the blood plasma of AD patients (Chiu et al., 2014).

According to Pansieri et al. (2018) findings, MNPs might be used to easily detect amyloidosis by imaging the amyloidogenic plaque or fibril depositions on the surface of the cell. Under optimal circumstances, this approach has been shown to be safe and non-toxic, according to the research. It is still necessary to explore the biocompatibility of free or functionalized MNPs in terms of medical significance, as well as their functionality. Nasr et al. (2018) tried to accomplish *in vivo* AD detection and developed magnetic NPs that could pass the BBB and detect the presence of A plaques. With the use of functionalized magnetic fields created by electromagnetic coils, Amin et al. (2017) devised a way for delivering FMNPs into normal mice brains. FMNPs shown the capacity to cross the BBB and reach the cortex and hippocampus of the brain. FeO MNPs coated with dextran containing osmotin were used to extend the effect to target Aβ in mice. It was shown to be beneficial in preventing synaptic loss caused by Aβ accumulation, expression of BACE-1 a, and other factors.

### Dendrimers

There are three elements to dendrimers: a central core, branches, and functional groups on the macromolecule’s surface. They take the form of a tree-like nanostructure. Macromolecular complexes containing nucleic acids or encapsulated drugs are effective because of their functional groups. To address the specific needs of the surface functional moieties, dendrimers are multivalent molecules that have a known structure and a fixed size (Mignani et al., 2013). With its hydrophilic functional groups, it has a lower viscosity than its linear polymer counterparts, and it is also more easily soluble in water. As a result, dendrimers may be engineered with a wide range of chemical modifications such as organic and inorganic groups attached to the branching site. Dendrimers are utilized in gene therapy in lieu of traditional viral vectors. When studied in mammalian cell types and animal models, dendrimers have showed encouraging outcomes. By endocytosis, it carries DNA into the nucleus, where it may be transcribed into the appropriate gene and product. Dendrimer-based treatment has an unusual benefit in that it does not stimulate the immune system (Lohan et al., 2017).

Another prominent polymeric carrier is dendrimer, which has gained favor in recent years as a means of gaining access to the cerebral cortex. Dendrimers have a number of benefits over other drug delivery systems, including high density, monodispersity, controlled size, and a high degree of surface functioning. These characteristics make them particularly well suited for site-specific drug administration. Many other kinds of dendrimers have been created using composite biomaterials like as PAMAM49, PPI50, carbosilane, PLL51, and triazine, among other things. Among the polymers used in biomedical applications, PAMAM is the most widely used and accepted because to its high biocompatibility, flexibility, and cheap cost (Igartúa et al., 2020). PAMAM dendrimer was administered with tacrine, an approved anti-AD medicine, according to Igartúa et al. (2020), with the goal of improving the therapeutic effectiveness of the therapy while simultaneously reducing the toxicity profile. Tacrine and dendrimers (DG 4.0 and 4.5) were mixed together in methanol, and the interaction between the two substances was investigated. The toxicological profile was examined using human RBC52 for *ex vivo* toxicity, cell culture experiments (Neuro-2a cell culture) for *in vitro* toxicity, and zebrafish larvae for *in vivo* toxicity. The results of the investigations were published in the journal Toxicology. When cells were treated with 300 micrograms of tacrine, the researchers discovered a substantial drop in cell viability. In contrast, when tacrine was delivered in conjunction with 1.8 mM of DG 4.0 or 4.5, no signs of toxicity were seen (Igartúa et al., 2020). They have previously used PAMAM dendrimer for carbamazepine brain delivery in order to increase drug solubility, decrease dosage and dosing frequency, lessen side effects, and lower the cost of treatment. Compaction with PAMAM dendrimer results in a large increase in the solubility of the medication, by almost thrice. Additionally, the formulation was proven to be safe in *ex vivo* human blood cell, *in vitro* N2a cell, and an *in vivo* zebrafish model (Igartúa et al., 2018). The brain targeting efficacy of Lf53 conjugated PAMAM dendrimers for administering memantine (NMDA antagonist) in an *in vivo* AD mice model was investigated in another comparable study. The 1HNMR confirmed that the Lf-PAMAM conjugation occurred as a result of a chemical process. The combination of Lf with PAMAM results in a considerable increase in the size of the drug-loaded PAMAM dendrimer, which rises from 11.5 to 131.7 nm. Since lactoferrin has cationic properties, it raises the zeta potential, which, when combined with surface coating, allows for slower drug release rates and longer durations of action than would otherwise be possible. The Lf conjugated dendrimer efficiently carried the medicine to the brain with enhanced bioavailability and shown improvement in memory and behavioral function in an AD animal model, according to the researchers (Gothwal et al., 2019). Another fascinating piece of research established the application of PEL54 dendrimer for brain delivery of flurbiprofen, an anti-inflammatory medicine licensed by the US Food and Drug Administration to treat AD. The dendrons were first created by solid-phase peptide synthesis and were then combined with flurbiprofen to create the final product. Mass spectroscopy and Fourier transform infrared spectroscopy were used to validate the interaction of the polymer and the medication. The formulation was tested for cell viability using bEnd.3 cells, and the results revealed that the system was very biocompatible. Incorporating the drug into the PEL dendrimer improved penetration through the BBB and hydrolysis of the drug transported the drug to the targeted spot.

### Quantum Dots

Peptides containing 39–42 amino acids, the majority of which are Aβ_1–40_ and Aβ_1–42_, are found in humans. In order to generate Aβ plaques, the abundance of Aβ_1–40_ and Aβ_1–42_ and their fibril-forming abilities lead to the creation of Aβ-peptides. AD is caused by the aggregation of Aβ peptides, mature fibrils, and soluble oligomers (Roychaudhuri et al., 2009). However, because of the accompanying toxicity and development of resistance, there are only a few authorized medications that can be used to treat AD (Gorain et al., 2020). Peptides, organic compounds, and synthetic peptides have showed promising outcomes in AD in preclinical investigations by removing aggregates or preventing the production of aggregates. Due to limited BBB penetration, a reduced *in vivo* stability and less effectiveness, they have no use for AD treatment (Takahashi and Mihara, 2008). Modern AD treatments use GQDs because of their capacity to suppress Aβ plaque development while simultaneously protecting cells from the harmful effects of A oligomers due to their tiny size (2–10 nm) and minimal cytotoxicity, making them very effective. Aβ plaques may be inhibited by hydrophobic interactions between carbon materials and A_β 1–42_ peptides, which lowers the negative surface potential and enhances the inhibitory efficacy of QDs (Mahmoudi et al., 2013; Liu et al., 2015c).

Tramiprosate linked covalently with GQDs inhibited A aggregation in AD in a synergistic manner (Mahmoudi et al., 2013). Glycine-proline-glutamate (Gly-Pro-Glu, GPE) was coupled with GQDs to generate GQDG nanomaterial, which inhibited the aggregation of A1-42 fibrils in APP/PS1 transgenic mice when intravenously delivered. With its tiny size and high surface area, GQDG can pass the MDCK cell monolayer and preferentially bind to the hydrophobic group A_β 1–42_ protein, which promotes their inhibition, as well as improve memory and learning capacity in mice receiving AD therapy (Xiao et al., 2016).

The unique properties of QDs allow them to overcome the limitations of conventional dyes and imaging techniques. Early detection of AD by the use of QDs is possible, since they can track the *in vivo* states of Aβ aggregation in mice in a variety of ways. Healthy mice and transgenic mice with human APP695swe and APP717 V–F mutations receive intra-cerebroventricular administration of fluorescent QD probes coupled with an anti-A antibody. Fluorescence microscopy and *in vivo* imaging indicated lower fluorescence intensity in APP transgenic mice compared to APP transgenic animals in the hippocampus, cerebral cortex, sagittal septum, and striatum of A1–42 fluorescent mice (Feng et al., 2013).

This ApoE-significant biomarker in AD might be detected electrochemically using CdSe@ZnS QDs as a sensing carrier. The immune-complex assay was used on a chip with flow mode to evaluate ApoE. AD biomarker tosyl-activated magnetic beads platform consists of a PDMS polydimethylsiloxane-polycarbonate microfluidic chip with integrated screen-printed electrodes. SqWave anodic stripping voltammetry demonstrated that CdSe@ZnS QDs had a lower detection limit and greater accuracy for diluted human plasma (Medina-Sánchez et al., 2014). The CdSe@ZnS QDs were manufactured by the same research group for the detection of apolipoprotein E (ApoE). For comparison research, Alexa 647 was used to assess the sandwich immunocomplex microarray. The experiment was carried out under the same circumstances as the standard enzyme-linked immunosorbent test (ELISA) targeting ApoE was used as a reference. In microarray assays, QDs have a lower limit of detection than Alexa microarray and ELISA, respectively, according to the findings of the excitation wavelength results (Morales-Narváez et al., 2012).

## Summary

Though administration of nanomaterials in *in vitro*, *in vivo* based preclinical studies showed significant improvement in the AD models, but still their clinical translation is somewhat lacking to demonstrate. There is a depressing truth that the first-line medications for clinical AD treatment only relieve symptoms but fail to prevent or reverse the course of AD. This is why so much time and money has been invested in understanding the pathophysiology of Alzheimer’s disease and creating viable treatment options. As a result of the disease’s many pathogenic variables and targets, including A, tau hyperphosphorylation, microglia, ROS, metal ions, and others, disease-modifying therapeutic methods are focused on a variety of approaches. In light of the failure of single-target treatments, it seems that multi-target combination therapies, which administer numerous medications simultaneously, have the brightest chances for treating AD. Nanomaterials have been shown to be capable of delivering many medications (e.g., chemical compounds, genes, peptides, and antibodies) at the same time, indicating a prospective use for the treatment of AD. There are some groundbreaking researches done in treatment or diagnosing AD using nanomaterials. For example, In Chinese patent, CN110559454B (2022) CRT (cathode ray tube) targeting peptides and QSH (quadrupole superparamagnetic ferrite) targeting peptides are used to modify a medicine-carrying nano micelle, which is used to transport an anti-amyloid protein and superparamagnetic ferrite medication for the treatment of Alzheimer’s disease. MRI imaging tracing and Alzheimer’s disease diagnosis and therapy are integrated into the nano composite medicine. The invention combines QSH targeting peptide and CRT targeting peptide to allow drug-loaded nano-micelles to pass through the blood-brain barrier by targeting both AD protein and transferrin. By targeting both AD protein and transferrin simultaneously, the concentration of a targeted site drug can be increased while the action time of the drug can be prolonged. In another patent, CN108685875A Anti-Alzheimer’s disease nano grain-pharmaceutical compositions are the subject of this invention, which focuses on preparations and applications in the pharmaceutical area (CN108685875A, 2022). Anti-AD combination treatments are now possible thanks to the present invention, which addresses a technological issue by extracting natural lipoproteins nanoparticles and AD medication recombination. Bio-imitability, safety, high drug load, efficient brain targeting, high affinity and targeting for amyloid protein are some of the features of the natural nano grain-pharmaceutical composition provided by the invention. The preparation conditions are mild, the process simple and the composition is easily industrialized. As a matter of fact, the patent CN110507830A, innovation focuses on a specific kind of nano-probe and its manufacture for Alzheimer’s disease pathogenic protein (CN110507830A, 2022). It’s a polyethyleneglycol derivative, as well as a phenothiazine derivative, that’s used to make the multi-modal nano-probe of the current invention. In the core of the nano-probe is an extra-small ferrite nanometer particle, and the outside layer is a polyethylene glycol segment that is connected with the phenothiazine derivative. In addition to its unique near-infrared fluorescent label enhancement effect and T1–T2 nuclear magnetic resonance image Contrast improved effect, the multi-modal nano-probe may be particular in combination with beta-amyloid protein patch. In addition to its compact size, superior biocompatibility, radiation-free properties, and a range of without possible neurotoxicity, the probe has a strong application potential in the early detection of Alzheimer’s disease.

Based on clinical trials, https://clinicaltrials.gov/ was assessed on dated June 16, 2022, it was found that 2852 clinical trials were registered as recruiting and not recruiting filter, however only 1 study NCT03806478 was registered as nanomaterial based technique (Mirani et al., 2017). APH-1105 NPs is investigational drug product which is sterile, pyrogen free lyophilized powder for nose to brain administration. However this study in phase 2, stage and is documented to complete in 2024. Based on above mentioned clinical settings it can be said that there is vast difference between preclinical and clinical translation. This can also be accounted based on nanotoxicity arising from nanomaterials that has not be regulated by government agency and there are no strict guidelines for it. As the clinical trials, has been missing these has been no FDA approved drugs for it.

## Future Directions and Conclusion

According to this study, nanotechnology may be used to fight AD. However, further *in vivo* studies are needed to assess the safety of nanotherapeutic methods. Studies on the pharmacokinetic and pharmacodynamic characteristics are needed before the medications are tested in humans. There are still many investigations that are restricted to *in vitro* or animal model studies. The goal of this study was to summaries current nanomedicine breakthroughs in the treatment of AD. CNS illnesses, such as AD, need more effective, non-toxic nanomedicine formulations. To summaries, nanomaterials have the potential to open up a world of possibilities for both current chemicals and new formulations, paving the path for the future development of innovative therapeutic interventions for AD. As a result of the failure of single-target medicines, multi-target combination therapies seem to provide the best hope for treating Alzheimer’s disease. Nanomaterials have been shown to be capable of delivering many medications (e.g., chemical compounds, genes, peptides, and antibodies) at the same time, indicating a possible therapeutic option for AD. Based on the crosstalk between distinct therapeutic targets, the rationale of several combination treatment techniques was examined in this study in order to give reference points for future drug design.

In theory, treating the fundamental cause of AD should lead to positive memory and cognitive enhancement, as well as a reduction in neurodegenerative damage. Although gene therapy seems to be a potential alternative treatment technique, it still has certain drawbacks especially *in vivo* due to a lack of selectivity, limited efficiency, and direct host exposure to the non-viral transport vector. However, even though *ex vivo* gene therapy may minimize the risk of these difficulties, it has a number of downsides, largely due to its delivery technique and more intrusive process. Clinical trials for AD have a reported failure rate of 99.6% (Cummings, 2018). This is thought to be owing to the many pathways involved in most studies and a lack of knowledge about the mechanisms at play. The key to establishing a treatment approach for AD is figuring out how the disease’s hereditary predisposition interacts with the disease’s downstream molecular process. The ideal success rate of immunotherapy is also disappointingly low, since most clinical studies were forced to stop owing to uneven outcomes or the onset of significant side effects. In addition, most active and passive immunotherapy aimed at mild-to-moderate AD, with limited benefit in cognition in more severe patients. However, immunotherapy is unable to restore cognitive function and cognitive decline that develops at a later stage of AD. The high surface-to-volume ratio and lipophilic characteristics of nano-based treatments, on the other hand, present a potential approach for drug delivery across the BBB. Nanoengineered systems have showed good physicochemical qualities, which have led to a number of studies reporting that NPs have been effectively changed to encapsulate highly antioxidant or anti-inflammatory bioactive substances into specified brain regions (Babazadeh et al., 2020). Toxicology studies have shown that nanoparticle formulations may be harmful, but they haven’t been able to establish this yet in human trials, which is why further research is needed (Sharifi et al., 2012; Mirani et al., 2017; Cummings, 2018; Babazadeh et al., 2020; CN110507830A, 2022). If they could afford it at all, most people would have had little choice but to look for more affordable treatment choices. It is thus determined that more study and pricing control are important to further examine and tap into the efficacy of each therapy method, as well as to evaluate the probable efficacies of combinatorial use of all three aspects against AD. There is a long way to go, but we are confident that fresh trials will lead to the discovery of a novel medication for AD that will eliminate the disease.

## Author Contributions

All authors listed have made a substantial, direct, and intellectual contribution to the work, and approved it for publication.

## Conflict of Interest

The authors declare that the research was conducted in the absence of any commercial or financial relationships that could be construed as a potential conflict of interest.

## Publisher’s Note

All claims expressed in this article are solely those of the authors and do not necessarily represent those of their affiliated organizations, or those of the publisher, the editors and the reviewers. Any product that may be evaluated in this article, or claim that may be made by its manufacturer, is not guaranteed or endorsed by the publisher.

## Footnotes

1
https://pubmed.ncbi.nlm.nih.gov/

2
https://www.sciencedirect.com/

3
https://www.scopus.com/

4
https://scholar.google.com/
